# Magnetic Field Effect on Coagulation Treatment of Wastewater Using Magnetite Rice Starch and Aluminium Sulfate

**DOI:** 10.3390/polym15010010

**Published:** 2022-12-20

**Authors:** Nomthandazo Precious Sibiya, Gloria Amo-Duodu, Emmanuel Kweinor Tetteh, Sudesh Rathilal

**Affiliations:** Green Engineering Research Group, Department of Chemical Engineering, Faculty of Engineering and the Built Environment, Durban University of Technology, Durban 4001, South Africa

**Keywords:** coagulation, magnetite, rice starch, magnetized coagulant, alum

## Abstract

The use of synthetic coagulants to reduce suspended particles from drinkable water and wastewater is prompting new issues because it poses many health and environmental risks. Hence, improving the coagulation process using sophisticated nanotechnology with a magnetic field (MF) for quick recoverability emerges as being useful. In this study, the effects of magnetite rice starch (MS) and aluminum sulfate (alum) were investigated at a constant dose (3 g or 3000 mg/L) using a Jar test (six beakers) as potential low-cost coagulants for industrial wastewater treatment. At a high magnification of 1000× and a surface pore size of 298 µm, scanning electron microscopy and energy dispersive X-ray (SEM/EDX) analyses were utilized to elucidate the morphology of the coagulants. Coagulation was performed at 150 rpm (quick mixing) for 2 min, and 30 rpm (slow mixing) for 15 min. Thereafter, samples were allowed to settle (10–60 min) with and without MF. The findings showed more than 65% contaminants removal (turbidity and TSS) and 30% chemical oxygen demand (COD) removal using alum while MS showed 80% contaminants removal (turbidity and TSS) and 50% COD removal. MS showed an increase of more than 3% in contaminants removal (COD, turbidity, and TSS) when exposed to MF. As a result, the use of MS together with MF in water and wastewater treatment is anticipated as an environmentally benign and effective coagulant.

## 1. Introduction

Rapid industrialization has brought several environmental problems as a result of wastewater produced from various industrial activities. Depending on the industry, wastewater may contain a variety of contaminants such as lactose, oils and greases, proteins, nitrogen, and phosphorus-rich compounds, sediments, other organic materials, and detergents [1,2,3,4,5]. When this wastewater is released into the ecosystem without adequate treatment, it poses a severe ecological concern with significant health and environmental consequences [1,6,7]. As a result, adequate treatment is essential to improve the ultimate quality of the effluent before final disposal [8,9]. The existing wastewater treatment system outlines the methods such as primary treatment (separation of suspended solids and large solid objects via screening), secondary treatment (reduction of dissolved solids and organic matter via basic activated sludge, coagulation, disinfection, and so on), and tertiary treatment (additional process to further remove nutrients) [2,8,10,11,12]. To achieve severe water quality regulations, coagulation has been often utilized to improve the quality of treated wastewater [1,13]. Coagulation/flocculation (CF) involves many stages, including the electrostatic interaction between negatively charged suspended impurities and cationic proteins, followed by particle destabilization [3,4,14,15,16,17]. Coagulants derived from chemicals such as (Al_2_(SO_4_)_3_ (alum), lime, and FeCl_3_ are often used in conventional CF processes due to their accessibility of use and low cost [18]. Nevertheless, the chemical-based coagulants were ruled inappropriate owing to the potential dangers and disastrous consequences on marine organisms [7,14,19,20,21,22,23,24]. Furthermore, they change the pH of the water, which might cause secondary pollution [25,26].The remaining aluminum particles in treated water have been connected to Alzheimer’s disease. [27,28,29,30]. Due to the drawbacks of these coagulants, there has been a quest for eco-friendly and sustainable coagulants for their usage. Natural coagulants are available locally, generate less sludge, and produce biodegradable sludge (which may be reused). They can also be waste-based resources such as fruit wastes, eggshells, cassava peel, okra leaves, and so on. [1,31]. In its unprocessed form, starch is one of the world’s most common natural polymers, composed of the polymers amylose and amylopectin, which are two anhydroglucose unit polymers [32,33]. Amylose is a non-branching helical polymer composed of −1,4 linked D-glucose monomers, whereas amylopectin is a polymer with several branches made up of −1,4 and −1,6 connected D-glucose monomers [34,35]. Due to its recyclability, biocompatibility, and low cost, starch has received commercial interest as a coagulant [32,34].

The advancement of nanotechnology has significantly aided in the development of novel approaches to numerous health and environmental challenges while using less energy [36,37]. Numerous research have been undertaken to establish magnetite’s efficacy: Fe_3_O_4_ (F) in the reduction of harmful metals and dangerous compounds from water and demonstrate the effectiveness of this material [38,39,40,41,42,43]. Fe_3_O_4_ can be used to coat a variety of surfactants to reduce aggregates induced by atomic magnetic interactions between molecules [44,45]. In terms of application, it has unique and favorable characteristics: cheap expense and danger, high magnetic, durability, bio-compatibility, and simplicity of dissociation from aqueous solution [44,46,47]. The fact that it is super paramagnetic, suggests that it responds to a magnet, making sample and collection easier and faster [48]. However, as the external magnetic field is removed, its magnetization diminishes. The employment of MF enhances settling capacities, decreasing the concentration of suspended particles and activated sludge, and boosting the wastewater management efficacy [49]. Previous research has demonstrated the efficacy of natural coagulants (rice starch, okra, eggshell, cassava leaves, banana peels, chitosan, moringa oleifera, etc.) via treating of turbidity through the neutralization of negatively-charged particles with positively-charged polymers [2,24,50,51,52,53]. Therefore, this study aims to efficiently investigate alum and rice starch incorporated with magnetite (MS) in wastewater treatment with and without MF. Lastly, scanning electron microscopy and energy dispersive X-ray (SEM/EDX) were utilized to investigate the elemental composition and the morphology of the coagulants under investigation.

## 2. Materials and Methods

### 2.1. Material

All of the reagents employed in the research were analytical graded and did not require additional purification, except for the stated ones and the deionized water (ELGA WATERLAB, PURELAB Option-Q water deionizer (High Wycombe, London, UK)) utilized in the stock solutions’ preparation. The chemicals utilized comprised NaOH pellets, FeSO_4_.7H_2_O, oleic acid (surfactant), ethanol, alum (Al_2_(SO_4_)_3_), FeCl_3_.6H_2_O, and the rest of reagents utilized in the preparation of synthetic wastewater were analytical grade and supplied by Sigma Aldrich (Kempton Park, South Africa). Rice starch was acquired from a local market (Shoprite, Durban, South Africa), washed with distilled water, and then oven dried for 24 h at 80 °C.

### 2.2. Synthetic Wastewater

At room temperature, the synthetic wastewater was made-up by mixing peptone (4 g), glucose (2.75 g), NaHCO_3_ (27.5 g), urea (0.75 g), meat extract (6.25 g), MgSO_4_.7H_2_O (0.05 g), K_2_HPO_4_ (0.7 g), CuCl_2_.2H_2_O (0.00125 g), NaCl (0.22 g), and CaCl_2_ (0.10 g) in 20 L of deionized water and 5 L of wastewater taken from a local wastewater treatment plant after chlorination process in eThekwini municipal area of South Africa [35]. The synthetic wastewater was evaluated for turbidity, TSS, and COD immediately after preparation, by APHA [54]. Turbidity was measured using a turbidimeter (Hach, 2100N (Loveland, Colorado)) while COD and TSS were measured using a spectrophotometer (HACH, DR 3900 (Düsseldorf, Germany)). The synthetic wastewater sample (0.2 mL) was measured, placed in the COD high range vials (HACH), and digested at 150 °C for 2 h. Following digestion, the bottles were cooled to 25 °C before the COD was measured using the spectrophotometer indicated above. The initial synthetic wastewater concentrations were measured in terms of turbidity (82.50 ± 1.105 NTU), COD (351.80 ± 0.781 mg/L), and total suspended solids (TSS; 77.33 ± 1.697 mg/L). While the received wastewater had an initial turbidity of 11.78 NTU, 6.5 mg/L TSS and 73 mg/L COD.

### 2.3. Synthesis of Magnetite Rice Starch (MS)

Firstly, the stock solutions of 0.4 M Fe^3+^ and 0.2 M Fe^2+^ were made by weighing 108.12 g and 55.61 g, respectively, and dissolving them in 1 L deionized water. In addition, a 3 M NaOH stock solution was prepared by dissolving 199.99 g of NaOH in 1 L of deionized water [35,55]. The magnetite (F) was then synthesized in a volume ratio of 1:1 by utilizing 500 mL of each of the Fe^3+^ and Fe^2+^ stock solutions. To achieve homogeneity, the solution was constantly agitated on a magnetic hotplate then 4 mL of oleic acid was added drop-wise. The pH of the solution was then adjusted to 12 with 250 mL of 3 M NaOH until a black precipitate appeared. The thick black precipitate was then heated (aged) at 70 °C. To remove any undesirable particles, the supernatant was decanted, and the precipitate was washed three times with distilled water and ethanol which was then oven drying at 80 °C for 12 h. After properly combining 20 g rice starch and 20 g magnetite (F), the magnetic rice starch (MS) was calcined in a Kiln Furnace (Cape Town, South Africa) at 550 °C for 1 h. The physical morphologies and elemental compositions of the coagulants (rice starch, alum, magnetite, and MS) were investigated at the University of Cape Town, South Africa, using scanning electron microscopy and energy dispersive X-ray (SEM/EDX) equipment (Nova NanoSEM paired with EDT and TLD detector).

### 2.4. Coagulation

**Figure 1:**
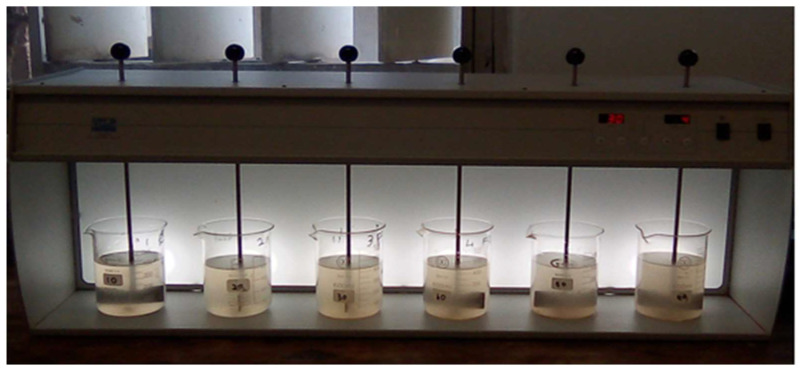
A jar test (JTL6) set-up for the coagulation/flocculation process.

Coagulation tests were performed at 25 ± 3 °C in beakers containing 500 mL of wastewater using a jar-test (VELP Scientifica, JTL6, Usmate Velate MB, Italy) as indicated in Figure 1. Each beaker was dosed with 3 g of coagulant at a contact pH (7.2) measured using Hanna pH-meter (Hanna Instruments, HI98130 (Durban, South Africa)). The impact of the settling period (10–60 min) with and without magnetics Field (MF) on pollutants elimination (turbidity, TSS, and COD) was also investigated. The beakers were then stirred swirled for 2 min at a fast-mixing rate of 150 rpm, followed by 15 min of flocculation at 30 rpm for 15 min [30,31] before being allowed to settle. The proportion of contaminants (COD, turbidity, and TSS) was determined using the Equation (1).

(1) $$E_{removal}\left(\%\right)=\frac{B_{A}-B_{B}}{B_{A}}\times 100$$

where *B_A_* and *B_B_* represent the initial and final values of each pollutant (TSS or turbidity or COD), and *E* is the % removal.

## 3. Results and Discussion

### 3.1. Scanning Electron Micrograph (SEM)/Energy Disperse X-Ray (EDX)

**Figure 2:**
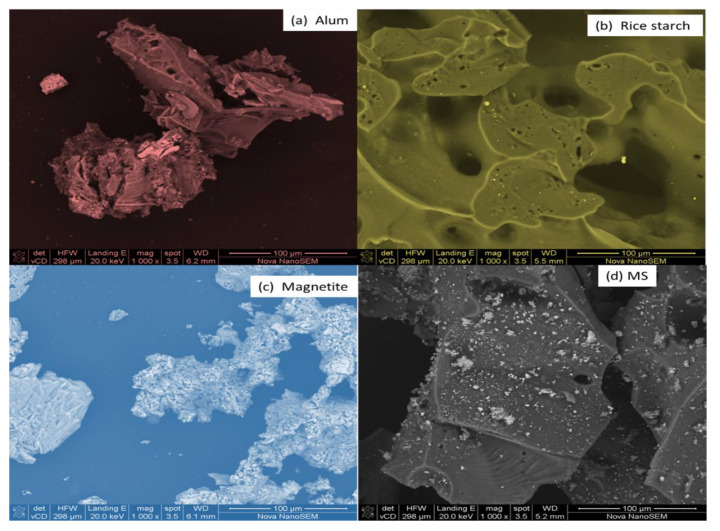
SEM images of coagulants before coagulation taken at a view field of 298 µm at a high magnification of 1000×; (**a**) alum, (**b**) rice starch, (**c**) magnetite, and (**d**) MS.

The magnetite, alum, rice starch, and MS were measured at a micro-scale of 100 µm, with a magnification of 1000×, a horizontal field width of 298 µm, and a landing energy capacity of 20 keV (Figure 2). Figure 2a–d depicts the SEM findings of alum, magnetite, rice starch, and MS obtained at working distances (WD) of 6.2, 6.1, 5.5 and 5.2 mm, respectively, ranging from large to low porosity, which may be owing to the high calcination temperature of 550 °C, which improved the wastewater adsorption capacity [36,55]. Alum (Figure 2a) image shows irregular-shaped particles in the middle of the surface produced by the agglomerations of various sizes of crystals. There are no obvious surface fractures, indicating that calcination did not generate dehydration [56]. Rice starch particles may be distinguished by particle size and granule shape [57,58].

Figure 2b demonstrated a smooth and solid surface with holes. The images of rice starch from this study coincide with the findings of Saritha et al. [58]. The crystallinity to carbohydrates might not have had a significant impact on water clarification efficacy. However, the characteristics of gluing that are related to crystalline phase level may have a good results on the removal of turbidity [59,60]. The magnetite (Figure 2c) exhibited a regular cellular structure that is similar to previous research [36,55]. Figure 2d shows an extreme filmed surface, at which MS revealed clusters with many pores. Furthermore, magnetizing rice starch had little influence on its shape since no depression, fractures, or hollow granules were found.

**Figure 3:**
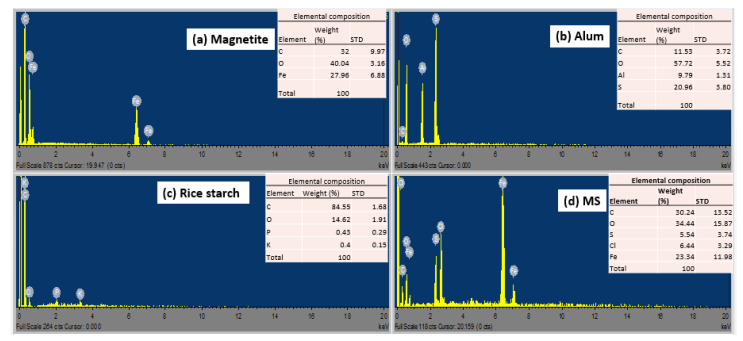
EDX images of (**a**) magnetite, (**b**) alum, (**c**) rice starch, and (**d**) MS.

Figure 3 shows the EDX micrograms of the coagulants studied. The magnetite spectra (Figure 3a) revealed an O (40.04%) > C (32%) > Fe (27.96%) composition, whereas the alum spectrum (Figure 3b) revealed an O (57.72%) > S (20.96%) > C (11.53%) > Al (9.79%) composition similar to previous study [36], and composition of C (84.55%) > O (14.62%) > *p* (0.43%) > K (0.40%) was found in spectra of rice starch (Figure 3c). The strong appearance of C & O peaks confirms the hypothesis that the rice starch granules are entirely composed of carbohydrate monomers [61,62]. Literature shows that magnetite is made up primarily of iron and oxygen [39,41,55,63]. Significant proportions of new components of S (5.54%), Cl (6.44%), and Fe (23.34%) were also discovered. MS spectrum (Figure 3d) reveals Fe binding energies at 0.6, 6.4, and 7 keV. Furthermore, the content of oxygen increased from 14.62 to 34.44%, whereas carbon content reduced from 84.55 to 30.24%. This confirms the effective synthesis of magnetized rice starch, which has improved the possibility of antibacterial and metal binding properties in various regions of the coagulant.

### 3.2. Comparative Study of Magnetic Effect Using MS and Alum

**Figure 4:**
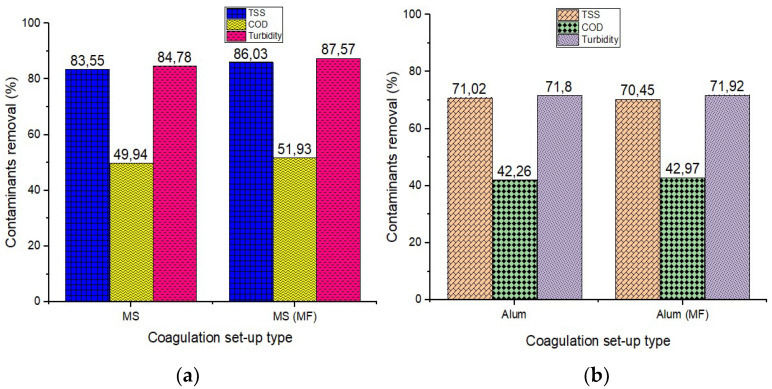
Effect of magnetic field using (**a**) MS and (**b**) alum on contaminants removal (%).

Figure 4 depicts the results of alum and MS treatments with and without MF (20 mT) over constant 60 min settling phase. Figure 4a shows that when a magnetic field was applied during the settling phase, turbidity, TSS, and COD efficiency increased from 84.78 to 87.57%, 83.55 to 86.03%, and 49.94 to 51.93%, respectively. The presence of magnetite in rice starch and MF led to higher treatability efficiencies and settle ability of the agglomerated flocs, and lower energy use [64,65]. The positively charged magnetite aided the adsorption of negatively charged pollutants in the wastewater sample [48], whereas the cationic exchange mechanism (electrostatic, van der Waals, and chemical bonding) and the generation of hydroxide on the surface of MS was used to reduce contaminants by magnetic measures [18,37,66]. Finally, amylase and amylopectin in rice starch destabilize colloidal particles by bridging and agglomeration [18,67,68]. As shown in Figure 4b, there is no significant/observable change in the use of the alum, when the magnetic field was applied. This might be due to lack of paramagnetic properties of the alum; whereas the magnetic entity of the MS (Figure 4a) facilitated its aggregation and settleability when the magnetic field was applied.

**Figure 5:**
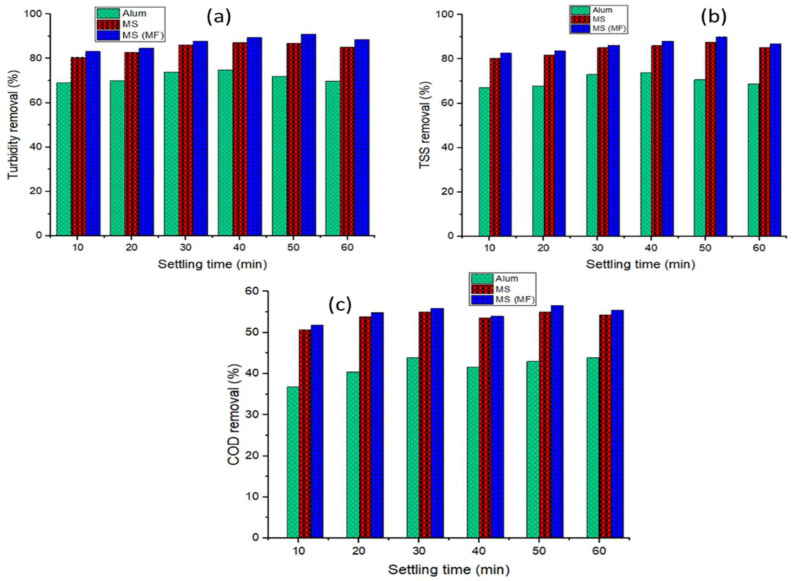
Results on (**a**) turbidity, (**b**) TSS, and (**c**) COD removal (%) with the effect of settling time.

It was observed that there is an intermolecular relationship between turbidity and TSS elimination (Figure 5) since they display a similar pattern [36,69]. Findings show that MS eliminated turbidity and TSS by more than 80% in both settling configurations, whereas alum eliminated turbidity and TSS by more than 70%. With settling durations ranging from 10 to 30 min, alum was found to enhance colloidal aggregation and destabilization (as demonstrated in the treatment of organic material and hydrophilic organic matter) for all contaminants (TSS, turbidity, and COD).

Colloidal stability and destabilization are produced by an enhancement in ionic strength with a slight decrease in zeta potential and a decrease in the thickness of the diffuse half of the electrical double layer [36,70,71,72,73]. Following that, the treatment ability of contamination (TSS and turbidity) became unstable. Since additional pollutants have been adsorbed, the flocs’ settling capacity has decreased as a result of a colloidal breakdown in suspension [37,74]. Due to general interaction between oxygen-containing groups (hydroxyl and carboxyl) and negatively charged colloidal particles, the surfaces may have become more hydrophilic (less hydrophobic) [36,75,76,77,78]. Figure 5c demonstrates that alum (>30 min) efficiency decreased at 40 min and then rose till the 60th min. The dramatic decrease in contaminants removal with time (30–40 min) for alum reflects the fact that the number of particles accessible for coagulation decreased as the reaction continued. This might be because of the flocs mechanism or the combination bridging mechanism [36,79]. Figure 5 suggests that extending the settling time improves removal performance for MS and MS (MF) until 40 min, at which point effectiveness begins to decline until 60 min. The residual contaminants decrease between 10 and 40 min resulting in the formation of large-size flocs with a greater settling velocity [80,81], but the trend almost reverses between 50 and 60 min, which could be due to colloidal entrapment and high attractive force dominants [12,36,75]. Although 40 min produced higher efficiencies, 30 min was chosen due to the quick and reasonable responses. As a result, the shorter the duration of settling, the more effective the procedure in terms of sludge reduction or management. The removal efficiency results for turbidity, TSS, and COD were recorded for each coagulant at an optimum settling time of 30 min (Alum: 74.03%, 73.80%, and 45.03%; MS: 86.25%, 85.09%, and 55.04%; and MS (MF): 87.95%, 88.24% and 55,97%). Table 1 shows different types of magnetized coagulants utilized in the treatment of various wastewaters.

## 4. Conclusions

Rice starch magnetite (MS) was produced utilizing a co-precipitation approach that combined rice starch with Fe_3_O_4_ in a 1:1 ratio. Analytical data obtained using scanning electron microscopy (SEM) in conjunction with energy-dispersive X-ray (EDX) spectroscopy confirmed the success of MS surface morphology and elemental compositions. The presence of multivalent ions (Fe, P, K, S, Cl Al) and their associated carbonates was verified by SEM/EDX, indicating a successful synthesis. The influence of settling time was explored in this research using alum and magnetite rice starch (MS) with and without exposure to a magnetic field (MF). At a settling period of 30 min with and without MF, the treatability performance of MS demonstrated more than 80% removal efficiency (turbidity and TSS) and more than 49% elimination of COD. MS (MF) has demonstrated a strong potential for water and wastewater treatment as an alternative to traditional alum. The addition of magnetite to rice starch resulted in faster particle aggregation and greater flocs size, this made quick settling possible. This finding implies that incorporating a magnetic field and magnetite rice starch into the coagulation process is a viable potential for wastewater treatment.

## Figures and Tables

**Table 1:** Different types of magnetized coagulants are used in the treatment of wastewaters.

Type of Coagulant/(s)	Effluent Source/(s)	Optimum Conditions	Contaminant Removal Efficiency	Reference/(s)
Magnetized eggshell	Synthetic wastewater	20 mg/L dosage 30 min settling	94.86% TSS 92.56% turbidity	[36]
Magnetized moringa oliferea	Surface raw water	400 mg/L dosage 30 min settling time	94.4% turbidity	[43]
	Textile wastewater	30 mg/L dosage 10 min magnetic exposure	91.43% turbidity	[46]
	Sanitation wastewater	10 mg/L dosage 30 min magnetic exposure	90% turbidity	[50]
Magnetized alum	Synthetic wastewater	20 mg/L dosage 30 min settling	99.75% TSS 99.50% turbidity	[36]
Magnetite rice starch (MS)	eThekwini municipal synthetic wastewater	3000 mg/L dosage, 30 min settling time	86.25% turbidity, 85.09% TSS, and 55.04% COD	This study
Magnetite rice starch (MS)	eThekwini municipal synthetic wastewater	3000 mg/L dosage 30 min magnetic exposure	87.95% turbidity, 88.24% TSS and 55.97% COD	This study
Magnetized alum	Textile effluent	50 mg/L 50 min magnetic exposure	85% turbidity	[37]

## Data Availability

Not applicable.
